# Multiplicity adjustment approaches in publicly funded multi-arm trials: a comprehensive review of the National Institute for Health and Care Research (NIHR) Journals Library

**DOI:** 10.1186/s13063-025-09324-5

**Published:** 2025-12-08

**Authors:** Ellen C. Lee, Richard M. Jacques, Rebecca M. Simpson, Stephen J. Walters

**Affiliations:** School of Medicine and Population Health, The University of Sheffield, Sheffield, UK

**Keywords:** Review, Multiple testing, Randomised controlled trial, Multi-arm trial, Type 1 error, Multiplicity

## Abstract

**Background:**

Parallel-group multi-arm trials are randomised controlled trials (RCTs) where participants are allocated to three or more concurrent treatment groups. Multiplicity occurs when several statistical tests are conducted within the same study. Statistical adjustments to the design and analysis of multi-arm trials can be used to control the study-wise type I error rate. There is no clear guidance or consensus on the necessity of multiplicity adjustment in multi-arm trials, nor on which methods are most appropriate. This comprehensive review aimed to investigate the design, analysis and reporting of publicly funded parallel-group multi-arm trials and to report the approach to multiplicity in these trials with respect to sample size and statistical analysis.

**Methods:**

We searched the United Kingdom’s National Institute for Health and Care Research (NIHR) online Journals Library, from 1 January 1997 to 31 December 2024 for reports of multi-arm RCTs. Information on the trial characteristics, the sample size estimation and analysis of the primary outcome was extracted. Two researchers conducted the search and selected reports for inclusion. Data from each report was independently extracted by two reviewers, and any disagreement was resolved by discussion.

**Results:**

A total of 2452 reports, published online in the NIHR Journals Library, were screened for eligibility; 97 reports of multi-arm parallel-group trials met the inclusion criteria. Of these, 90 included the results of a multi-arm efficacy analysis. In the review, 35% (34/97) of the trials did adjust for multiplicity in the sample size calculation; in 84% (76/90), the potential between-arm comparisons were described in the methods, and 37% (33/90) made a multiplicity adjustment in the analysis. A further 86% (77/86) reported 95% confidence intervals. For the minority of multi-arm trials that did adjust for multiplicity, the most common adjustment method was Bonferroni.

**Conclusions:**

The majority of the publicly funded multi-arm trials did not adjust for multiplicity in the sample size, statistical analysis, or estimation of confidence intervals. Researchers should follow the Consolidated Standards of Reporting Trials guidelines for multi-arm trials and clearly state in protocols and trial reports whether a multiplicity adjustment was made or provide a reason if no adjustment was made.

**Supplementary Information:**

The online version contains supplementary material available at 10.1186/s13063-025-09324-5.

## Background

Parallel-group multi-arm trials are randomised controlled trials (RCTs) where participants are allocated to three or more concurrent treatment groups. A review by Pike et al. [1] found 17% of late phase RCTs published in 2018 were multi-arm trials. They are generally considered more efficient than two-arm trials; for example, a single control group can be used to compare against multiple new treatments, or multiple regimens of the same treatment, saving time and cost compared to conducting several two-arm trials. A trial with multiple treatment arms can also increase the likelihood of finding a new treatment that works within a single trial [2]. However, these benefits come at a potential cost of statistical complexity due to multiple treatment comparisons. Multiplicity occurs when several statistical tests are conducted within the same study; this can increase the probability of a type I error (a false positive), which can result in incorrectly recommending a new treatment.

Statistical adjustments to the design and analysis of a multi-arm trial can be used to control the study-wise type I error rate. These might typically include using hierarchical or ordered testing, or adjusting the significance level for inference and the corresponding confidence intervals for each test, alongside inflating the sample size. There is no clear guidance or consensus on the necessity of multiplicity adjustment in multi-arm trials, nor on which methods are most appropriate. The CONSORT (Consolidated Standards of Reporting Trials) extension for multi-arm parallel-group trials [3] acknowledges this, stating ‘the decision regarding [multiplicity] adjustment depends on the objectives, design, and analysis’. The extension does however specify reports should ‘explicitly state if no adjustments for multiplicity were applied; if adjustments were applied, state the method used’ and that reports should include ‘results for each prespecified comparison of treatment groups’.

There have been several published reviews of multi-arm trial design. Pike et al. [1] reviewed 23 publicly funded trials with three or more treatment groups published in 2018. They found variation in practice, noting their findings suggested researchers were more likely to adjust for multiplicity when comparing related treatments than when comparing distinct treatments (9/15 trials comparing related treatments adjusted, 2/8 comparing distinct treatments adjusted). Odutayo et al. [4] reviewed 64 multi-arm trial protocols approved by research ethics committees in 2012. Of the 50 protocols that involved multiple testing, 28 used adjustments to control the type I error rate (nine using a single step procedure, 17 using an ordered sequence/hierarchical testing). They also found discrepancies with the subsequent results publication. They concluded that strategies to reduce the type I error in multi-arm trials are inconsistently employed and important differences existed between planned analysis and subsequent publications. Wason et al. [5] reviewed 59 multi-arm trials published in 2012 and found nearly half (49%) included a multiple testing correction. They also found the proportion that corrected was higher for trials that investigate multiple regimens or doses of the same treatment (67% adjusted). Baron et al. [6] found 60% of trials published in 2009 (180/298) described planned pairwise comparisons, 11% of which did not report these pairwise comparisons. They also found that of the 204 articles that reported pairwise comparisons, these comparisons were not planned in 44 cases (22%).

The motivation for conducting this review was to investigate the design and reporting of multi-arm trials, with particular focus on the approach to multiplicity. Our interest was in publicly funded trials, for which the research hypotheses can be more diverse and not necessarily subject to regulatory guidelines. The National Institute for Health and Care Research (NIHR) is funded through the UK government Department of Health and Social Care. NIHR publishes comprehensive accounts of its funded research within its online Journals Library. These include detailed description of methods and have ample space for justification of choices to be included. Publication bias is likely to be low as NIHR publish reports of all their funded research, and all projects have had their design scrutinised by a panel of experts, so the research will be of high quality. The Journals Library comprises six open access peer-reviewed journals reporting results from a range of health research areas: Health Technology Assessment (HTA) [7] published its first volume in 1997, Health and Social Care Delivery Research (HSDR) [8], Programme Grants for Applied Research (PGfAR) [9], and Public Health Research (PHR) [10] journals published their first volume in 2013. Efficacy and Mechanism Evaluation (EME) [11] published its first volume in 2014, and Global Health Research (GHR) [12] in 2024.

This review aims to investigate the design, analysis, and reporting of parallel-group multi-arm trials funded by the NIHR and to report the approach to multiplicity in these trials with respect to sample size and statistical analysis.

## Methods

### Search strategy and trial identification

We manually searched all online articles published in the six journals of the NIHR Journals Library between 1 st January 1997 and 31 st December 2024. Title and abstract were screened to ascertain if a parallel-group multi-arm RCT was reported; if information in the title and abstract was insufficient to determine if a trial was eligible, the rest of the report was searched. The reports were obtained from the NIHR Journals Library website [13]. Two researchers conducted the search (RMJ searching articles published up to 2023, ECL 2023 onwards) and selected reports for inclusion. If the inclusion of a trial was in doubt, this was discussed by all authors.

### Eligibility criteria

Eligible articles were reports of multi-arm parallel-group randomised controlled trials published in any of the six online journals of the NIHR journals library between 1 st January 1997 and 31 st December 2024. Reports on all non-trial designs and pilot/feasibility trials were excluded. Likewise, adaptive designs were excluded as these studies allow for prospectively planned modifications to trial design. Crossover and factorial trials were excluded as these have their own design considerations. Multi-arm trials that stopped early and did not perform any efficacy analysis or trials that had unplanned dropping of treatment arms to become a two-arm trial were included in the design summaries only.

### Data extraction

Once the NIHR reports had been selected for inclusion, information was extracted from each report using a data extraction form (Excel spreadsheet) that had been piloted on 5 reviews. Data extraction was undertaken by a team of reviewers (RMJ, ECL, RMS, SJW). Data from each report was independently extracted by two reviewers, and any disagreement or uncertainty was resolved by discussion.

The following information was extracted for each trial:

- Trial characteristics, including trial design, unit of randomisation, clinical area, setting, trials unit involvement, number of arms, allocation ratio, trial hypothesis, intervention types, patient blinding, inclusion of pilot, geographical region, primary outcome
- Sample size, including any revision to sample size, power and alpha used in sample size calculation, any method of alpha adjustment in sample size, and if so, details of adjustment
- Analysis, including approach to multiplicity in statistical analysis, potential and actual number of primary comparisons, p-value adjustment, confidence interval nominal coverage level

Each reviewer assessed whether they thought trial treatment arms were ‘definitely related’, ‘probably related’, ‘distinct’ or ‘unsure’. After extraction, one reviewer (ECL) re-categorised all studies into the following criteria based on extracted information and the two original reviewers’ relatedness assessments.

- Closely related interventions vs control (e.g. personalised diet advice vs non-personalised diet advice vs control)
- Distinct treatments vs control (e.g. steroid injection vs physiotherapy vs control)
- Intervention groups combine intervention elements (e.g. paracetamol vs ibuprofen vs paracetamol and ibuprofen)
- Interventions could be similar/some similarity (e.g. GP-led telephone triage (GPT) vs nurse-led computer-supported telephone triage vs usual care)
- One intervention against multiple control groups (e.g. Group art therapy vs activity group (attention control) vs usual care)

### Analysis

Descriptive statistics on the study characteristics were summarised for the whole dataset and for trials reported after 2019 (when the CONSORT multi-arm extension was published [3]). Cross tabulation and graphs were used to describe relationships in the data. Descriptive statistics using frequencies and percentages were summarised for categorical trial characteristics, while range, median and interquartile range were obtained for continuous data. All analyses were performed in Stata v18 [14]. This study has been reported according to the Preferred Reporting Items for Systematic Reviews and Meta-Analyses (PRISMA) checklist [15] where appropriate.

### Patient and public involvement

Patients and the public were not involved in the design, conduct, reporting or dissemination plans of this research.

## Results

### Screening

**Fig. 1:**
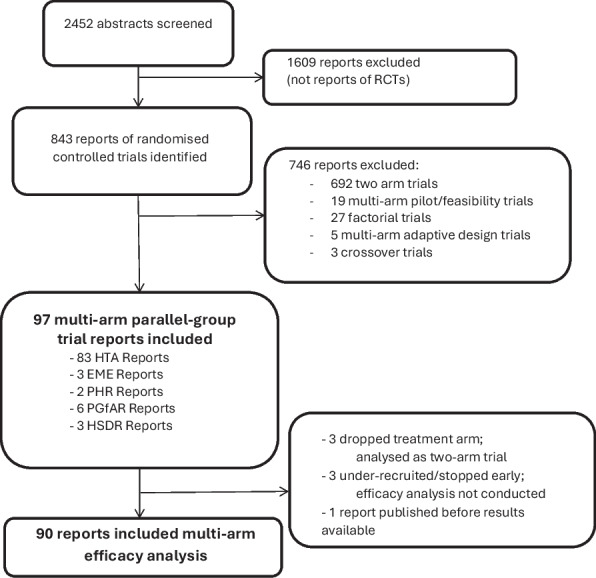
Flow diagram showing the search and selection process of RCTs from the six online journals of the NIHR Journals Library surveyed from 1 st January 1997 to 31^st^ December 2024

The search and selection flow diagram is presented in Fig. 1. Between 1 st January 1997 and 31 st December 2024, 2452 reports were published within the NIHR Journals Library. The search identified 843 articles reporting trials; 121 of these were reports of multi-arm trials. A further 5 reports were excluded due to reporting adaptive designs and 19 reports were excluded due to reporting pilot/feasibility multi-arm trials.

Three articles reported two three-arm trials with common design characteristics delivered in two strata/populations—each of these articles is presented as a single study in the results section, where any differences in approach or design were identified; the first trial is reported.

### Trial characteristics and sample size

Table 1 summarises the characteristics of the 97 multi-arm trials included in this review. The majority of trials were funded by HTA (86%), were individually randomised (88%) and had three arms (89%). Table 2 summarises the primary outcomes and sample size calculations used in the trials. The median sample size was 642, and over half of the trials had target sample sizes designed to achieve ≥ 90% power. Thirty-four (35%) trials adjusted the sample size calculation to account for multiple treatment comparisons. Of these, the most common adjustment was Bonferroni (*n* = 24), four trials used a threshold of 0.01, and three trials used a Dunnett adjustment. A further three trials stated adjustment had been made but did not describe the method used.

**Table 1:** Characteristics of the multi-arm trials included in this review (*N* = 97)

Characteristic	*N* (%)
	(*N* = 97)
Journal	
EME	3 (3%)
HSDR	3 (3%)
HTA	83 (86%)
PGfAR	6 (6%)
PHR	2 (2%)
Unit of randomisation	
Cluster	12 (12%)
Individual	85 (88%)
Setting	
Community	10 (10%)
General practice	29 (30%)
Hospital	41 (42%)
Mixed	12 (12%)
School	2 (2%)
Specialist services	3 (3%)
Trial design	
Parallel	89 (92%)
Two parallel trials/strata	6 (6%)
Trial with patient preference	2 (2%)
Number of treatment arms	
3	86 (89%)
4	7 (7%)
5	4 (4%)
Uneven allocation ratio	11 (11%)
Clinical Trials Unit^a^ involved	41 (42%)
Trial hypothesis	
Equivalence	1 (1%)
Non-inferiority	5 (5%)
Superiority	85 (88%)
Superiority and equivalence	2 (2%)
Superiority and non-inferiority	4 (4%)

^a^Clinical Trials Units are specialist units that design, conduct, analyse and report trials

**Table 2:** Summary of primary outcomes and sample size calculations used in multi-arm trials

	Summary	
	(*N* = 97)	
Co-primary outcome	21 (22%)	
Primary outcome(s) type		
Continuous	57 (59%)	
Binary	20 (21%)	
Time to event	9 (9%)	
Count	3 (3%)	
Ordinal	1 (1%)	
Multiple outcomes, different types	6 (6%)	
Percent	1 (1%)	
Timepoint of primary outcome		
≤ 1 month	14 (14%)	
> 1 and ≤ 6 months	30 (31%)	
> 6 and ≤ 12 months	26 (27%)	
> 12 months	18 (19%)	
No fixed timepoint	9 (9%)	
Original target sample size		
*N* (%)	96 (99%)	
Median (IQR)	642 (440, 1200)	
Min., max	87, 21,138	
Target sample size (including recalculations)		
Median (IQR)	600 (358, 1200)	
Power used in sample size calculation		
0.8	39 (40%)	
0.89	1 (1%)	
0.9	46 (47%)	
> 0.9	5 (5%)	
Not stated	3 (3%)	
Power given as a range	3 (3%)	
Alpha adjustment in sample size calculation		
No	58 (60%)	
Not reported	3 (3%)	
Not clear	1 (1%)	
Yes—for multiple primary outcomes only	1 (1%)	
Yes	34 (35%)	
If yes, sample size adjustment method^a^	*α* = 0.01	4 (4%)
	Bonferroni	23 (24%)
	Bonferroni, none in sample size recalculation	1 (1%)
	Dunnett	3 (3%)
	Not clear	3 (3%)

^a^Adjustment for multiple treatment comparisons only, one additional trial used Bonferroni for multiple primary outcomes

### Types of intervention

For 51 (53%) trials, the two allocated reviewers agreed the interventions were either ‘definitely related’ or ‘probably related’; for nine trials, the reviewers agreed the interventions were distinct, but for 37 (38%) trials, the reviewers were either unsure or did not agree on the relatedness of trial treatments (Table 3), suggesting it was difficult to determine the relatedness of interventions. There were six multi-arm trials that assessed one intervention against multiple control groups and 10 trials where intervention arms combined intervention elements (for example, paracetamol alone vs ibuprofen alone vs paracetamol plus ibuprofen). The most common multi-arm trial types were trials of either closely related interventions or interventions that had some similarity.

**Table 3:** Summary of trial interventions for the trials included in the review

Intervention details	*N* (%)
	(*N* = 97)
Active intervention type	
Behavioural/lifestyle/education	28 (29%)
Drug	24 (25%)
Equipment/device	10 (10%)
Physical activity/physiotherapy	5 (5%)
Procedure/surgery	8 (8%)
Service level intervention	7 (7%)
Speech therapy	2 (2%)
Other	13 (13%)
Control type	
Active	84 (87%)
Placebo	13 (13%)
Reviewers’ independent assessment of relatedness	
Agreed—definitely related	25 (26%)
Agreed—probably related	26 (27%)
Agreed—distinct	9 (9%)
—Did not agree/unsure	37 (38%)
Design type (EL assessment)	
Closely related interventions vs control	40 (41%)
Distinct treatments vs control	16 (16%)
Intervention groups combine intervention elements	10 (10%)
Interventions could be similar/some similarity	25 (26%)
Multiple control groups	6 (6%)

### Multiplicity adjustment approaches used

The majority of trials (*n* = 76, 84%) described the potential treatment comparisons in the methods section, and most of these trials planned to make two (37%) or three (38%) pairwise comparisons (Table 4). Thirty-three trials (37%) stated a multiplicity adjustment method would be employed; the most common of these being Bonferroni (*n* = 16) and hierarchical testing (*n* = 11).

**Table 4:** Summary of multiplicity adjustment approaches in analysis methods and results sections of the trial reports included in this review

Characteristic	*N* (%)	*N* (%)
	(*N* = 90)	
Potential comparisons described in methods		
No	14 (16%)	
Yes	76 (84%)	
If yes, number of potential comparisons	1	1 (1%)
	2	33 (37%)
	3	34 (38%)
	4	4 (4%)
	6	3 (3%)
	10	1 (1%)
Actual comparisons reported/undertaken		
1	3 (3%)	
2	41 (46%)	
3	39 (43%)	
4	5 (6%)	
6	1 (1%)	
10	1 (1%)	
Multiplicity adjustment^a^		
No adjustment, no reason provided	10 (11%)	
No adjustment, reason provided	13 (14%)	
Not mentioned	34 (38%)	
Adjustment made	33 (37%)	
Adjustment method	Alpha of 0.01^b^	2 (2%)
	Bonferroni	16 (18%)
	Dunnett	3 (3%)
	Hierarchical	11 (12%)
	Bonferroni (adjustment made to non-inferiority limit)”	1 (1%)
Reason for multiplicity adjustment		
Multiple outcomes	3 (3%)	
Multiple treatment groups	32 (36%)	
Multiple treatment groups and multiple outcomes	3 (3%)	
Analysis included global statistical test	19 (21%)	
Adjusted *p*-value in results		
Yes	15 (17%)	
No	65 (72%)	
Not reported	9 (10%)	
Not clear	1 (1%)	
Adjusted confidence interval in results		
Yes	11 (12%)	
No	75 (83%)	
not reported	4 (4%)	
Reported confidence interval nominal coverage level		
95%	77 (86%)	
97.5%	6 (7%)	
98.3%	2 (2%)	
99%	1 (1%)	

^a^Due to multiple treatment comparisons only, two further trials adjusted due to multiple primary outcomes using Simes (1) and Bonferroni (1)

^b^One trial also used hierarchical testing in addition to alpha of 0.01. The denominator (*N* = 90) reflects all reports that included multi-arm efficacy analysis, see Fig. 1 for details

Table 5 summarises the use of multiplicity adjustment by our reviewer-assessed design types. The cell counts are low, but there is no clear relationship between design type and choice of adjustment; 12/36 (33%) trials investigating closely related interventions adjusted, which is not dissimilar to the other design types.

**Table 5:** Presence/absence of multiplicity adjustment by trial design type

	Design type					
	Closely related interventions vs control	Distinct treatments vs control	Intervention groups combine intervention elements	Interventions could be similar/some similarity	Multiple control groups	Total
*N* (%)						
Multiplicity adjustment						
Adjustment made	12 (33%)	7 (47%)	4 (40%)	7 (29%)	3 (60%)	35 (39%)
No adjustment, no reason provided	1 (3%)	3 (20%)	2 (20%)	4 (17%)		10 (11%)
No adjustment, reason provided	7 (19%)			6 (25%)		13 (14%)
Not mentioned	16 (44%)	5 (33%)	4 (40%)	7 (29%)	2 (40%)	34 (38%)
Total	36	15	10	24	5	90

The denominator (*N* = 90) reflects all reports that included multi-arm efficacy analysis, see Fig. 1 for details

#### Reason for no adjustment

Of the 23 trials that stated in the methods that no multiplicity adjustment would be made, 13 gave a justification for this choice (Table 8 in the Appendix). The justifications made were:

- Three trials chose a single primary treatment comparison and considered all other treatment comparisons as ‘secondary’
- Two trials included a reference only
- Three trials argued that sample size adjustment was sufficient
  ◦ One of these left the significance level up to readers’ discretion
- One trial argued adjustment was not necessary as they carried out two primary comparisons
- One trial argued adjustments were unnecessary because a priori hypotheses were specified
- One trial underrecruited so stated their focus was on effect size and confidence intervals
- One trial assessed an equivalence hypothesis
- One trial tested two hypotheses via one model using two orthogonal contrasts

**Table 6:**
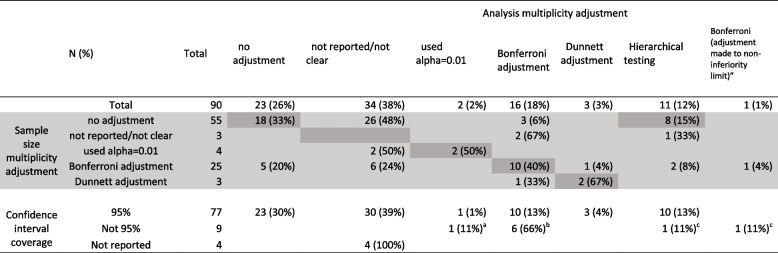
Sample size adjustment and confidence interval nominal coverage level by analysis multiplicity adjustment approach

Adjustments presented are those that adjusted due to multiple treatment comparisons only, two further trials adjusted due to multiple primary outcomes using Simes (1 trial—analysis only) and Bonferroni (1 trial, both sample size and analysis); the trials are included in this table as not adjusting

^a^99% CI

^b^Four trials presented 97.5% CIs, two trials presented 98.3% CIs

^c^97.5%

#### Consistency

**Fig. 2:**
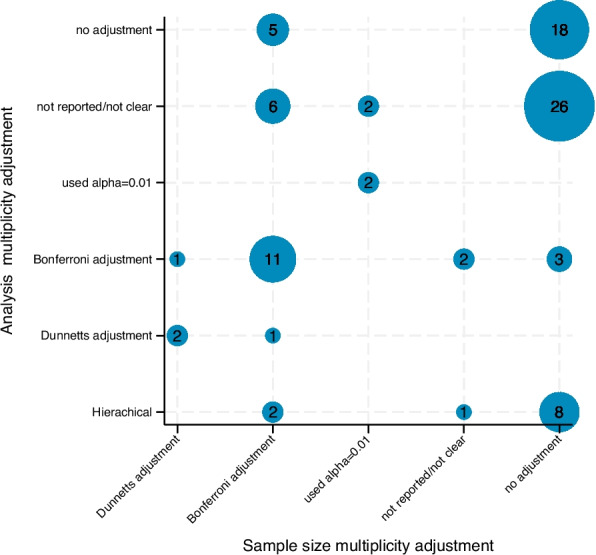
Consistency between multiplicity approach used in sample size calculation and statistical analysis (*N*=90)

It is not possible to fully assess the consistency between trial sample size calculation and analysis methods (Fig. 2, Table 6) due to incomplete reporting: Three trials did not report sample size, *p*-value or multiplicity adjustment method, and 34 trials did not describe analysis multiplicity adjustment method (or it was unclear) in the methods or results section. What is apparent from Fig. 2 is that there was not clear consistency between the approaches used in the sample size and analysis. For example, of the 25 trials that used Bonferroni adjustment in the sample size, fewer than half of these (10, 40%) also used Bonferroni adjustment in the analysis. Three trials did not adjust their sample size but did a formal alpha adjustment in the analysis (Bonferroni). Conversely, five trials that did not adjust their analysis included Bonferroni adjustment in their sample size calculation. There were 14 trials that included a multiplicity adjustment in the analysis but did not reflect this in the presented confidence intervals for treatment differences, instead choosing to present 95% CIs (Table 6).

### Reporting compared to the CONSORT multi-arm extension

There are three items in the CONSORT multi-arm extension that directly relate to multiplicity adjustment methods [3]. The statement recommends reports should include ‘planned sample size with details of how it was determined for each primary comparison’. This was not explicitly extracted in our review; however, we can infer that at least three trials did not meet this, as their multiplicity adjustment/alpha threshold was either not reported or not clear (Table 2).

In the statistical methods section, reports should ‘explicitly state if no adjustments for multiplicity were applied; if adjustments were applied, state the method used’ [3]. Thirty-four (38%) reports did not meet this criterion (Table 4).

Results reporting should include the ‘results for each prespecified comparison of treatment groups’. We collected the number of potential comparisons outlined in the methods and the number of actual comparisons reported in the results. Seventy-six (84%) trials described the potential comparisons in the methods section. Six trials presented more treatment comparisons in the results than they described (as potential comparisons) in the methods. Eleven trials presented fewer treatment comparisons in the results than they described in the methods; six of these had preplanned hierarchical testing.

### Approach and reporting over time

**Fig. 3:**
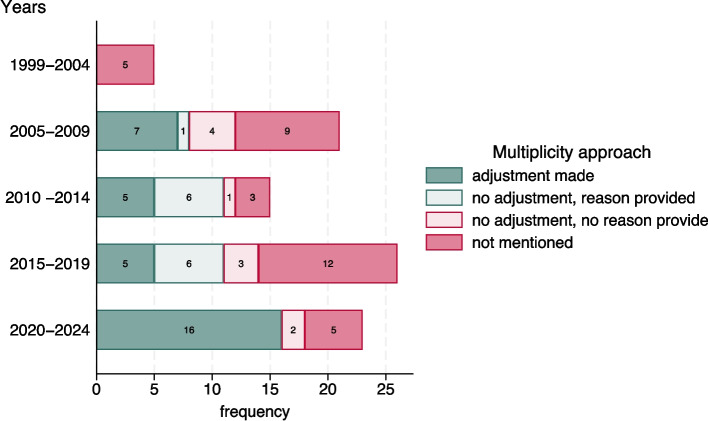
Multiplicity adjustment approach over time for the multi-arm trials included in this review (*N* = 90)

Twenty-three trials were published after 2019, the year the CONSORT multi-arm extension [3] was published (Appendix Table 7, Fig. 3). Hierarchical testing appeared more commonly used (7 out of the 11 trials that planned hierarchical testing were post 2019). A higher proportion of trials post 2019 reported a multiplicity adjustment plan (either adjusting or stating no adjustment); 78% (18/23) compared to 55% (26/65) of trials published in ≤ 2019. Sample size adjustment was clearly reported for all but one trial (although this is a similar prevalence to ≤ 2019 reporting).

## Discussion and conclusion

This study provides a comprehensive review of the design and analysis of 97 multi-arm parallel-group trials published by the UK NIHR between 1997 and 2024. The included trials are of high quality, having had their research proposals scrutinised by a panel of experts and external reviewers prior to funding approval. As the NIHR intends to publish all research it funds, this review has a minimal chance of publication bias compared with a review of other journals where publishing could be more selective. The NIHR journals include extended research articles that provide a full, single account of the funded research, allowing sufficient space to give details and justification on the multiplicity approach.

The trials in this review were most commonly three-arm (89%) and investigated either closely related interventions or interventions that had some similarity. Just over a third (35%) of trials included a formal adjustment to the sample size to account for multiple treatment comparisons, the most common approach being Bonferroni (25%). This was more than observed by Baron et al. [6] who found the multi-arm design was reflected in the sample-size calculation of 20% (41/210) of trials in their review.

The majority of trials (84%) described the potential treatment comparisons within the methods section, most planning on making up to two (37%) or three (38%) pairwise treatment comparisons. Thirty-seven percent of trials stated a multiplicity adjustment method would be used, most commonly Bonferroni or hierarchical/ordered testing. This is similar to the rates observed by Pike et al. [1] (39%), who also reviewed publicly funded trials, and by Baron et al. [6] (40%), but lower than that observed by Wason et al. [5] (49%) and Odutayo et al. [4] (50% of protocols reviewed planned adjustment). This could be due to multiplicity adjustment being more prominent in industry-funded trials. Both Wason et al. and Odutayo et al. also found hierarchical/ordered testing to be more commonly applied than a single-step multiplicity adjustment.

There was no clear relationship between choice of multiplicity adjustment approach and type of interventions under investigation. This is surprising as both Wason et al. [5] and Pike et al. [1] found adjustment was more frequently applied in trials where the experimental arms were related. It was difficult to determine if investigational treatments were related for some trials in this review, as this could be subjective and could require clinical expertise. This subjectivity was not reported in Pike et al. or Wason et al.

Thirteen (14%) trials in this review stated no adjustment would be made and provided an explanation for this choice. This is higher than the 3% of trial reports that gave justification in Baron et al. [6] and the 6% of protocols that gave a defence for not adjusting in the review by Odutayo et al. [4]. We have not commented on the suitability or merit of the justifications used in the trials in our review, but we note that including more than a reference is desirable. It was interesting that none of these trials used independence of treatments as a justification for not formally adjusting, as this is regularly argued as an appropriate context for non-adjustment in the literature [16, 17].

There was inconsistency across the multiplicity adjustment approaches employed in the sample size and subsequent statistical analysis, which was further complicated by unclear reporting for around a third of the trials in the review. Eight trials adjusted for multiplicity in the sample size or analysis alone. Of 21 trials that did include adjustment to the *p*-value in the results section, 14 chose to report 95% confidence intervals, whereas seven modified the confidence interval nominal coverage level to reflect the alpha adjustment.

Reporting was improved after the CONSORT multi-arm extension [3] was published; a higher proportion of trials published after 2019 stated their approach to multiplicity due to multiple treatment comparisons.

The study had several limitations. The review was limited to one UK-based funder, NIHR, and so does not necessarily reflect all publicly funded trials, nor can it be generalised internationally. Data was double extracted by two independent reviewers, but it is possible that errors have occurred. It is also possible that statements relating to multiple testing and the research questions were missed as our search focus was on the statistical methods and results section, although the discussion sections were also searched. We did not record the research hypotheses or research questions which could have helped with the interpretation of the appropriacy of the statistical approaches to multiplicity. For over a third of trials (38%), the two reviewers were either unsure of or did not agree on the relatedness of the interventions. This highlights the potential subjective nature of the assessment and the need for clinical/specialist insight on the interventions. The re-categorisation of design type also included some subjective assessment.

This review was predominantly historic, including trials with results published from as early as 1999 and excluding ongoing NIHR trials. Hence, we cannot expect it to reflect current standards and recommendations on the reporting and statistical conduct of trials that investigate multiple concurrent treatments. Further work should investigate a wider cross section of ongoing or recently completed trials to investigate the types of multi-arm trials that are currently undertaken and the approaches to multiplicity used in these trials; it could also expand to include other trial designs with multiple treatment comparisons such as factorial and platform trials.

This review found that multiple testing adjustment for multiple treatment comparisons is not applied in the majority of publicly funded multi-arm trials. It concurs with the findings of Baron et al. [6], who found ‘discrepancies between planned and reported comparisons’. We also agree with their sentiment that “reasons for using adjustment or not are often subjective and should be justified”, which was rarely done in the trials in this review: the most common ‘approach’ to multiplicity was not to mention multiplicity at all (38%) so there is clear potential for improvement in this area.

We agree with Molloy et al. [17] that clearer guidance from stakeholders on the appropriate setting for multiplicity adjustments would be beneficial. Formal statistical adjustment is unavoidable in some contexts, as regulators such as the European Medicines Agency require it [18], but there are contexts where no adjustment is considered acceptable by the research community, particularly when comparing multiple distinct treatments to control [16]. The recent popularisation of platform trials that require decisions regarding multiplicity related to multiple treatment arms makes this research all the more timely.

It is important to clearly report multi-arm trials, including a justification for the chosen multiplicity approach: Gaps in reporting and lack of justification for the sample size and analysis strategy may have implications for the interpretation of treatment efficacy and trial results.

## Supplementary Information

Supplementary Material 1.

Supplementary Material 2.

## Data Availability

Data is available online DOI: 10.15131/shef.data.29391680.

## Appendix

**Table 7:** Further characteristics of the multi-arm trials included in this review (*N*=97)

Characteristic	*N* (%)
	(*N*=97)
Publication year	
1999	1 (1%)
2000	1 (1%)
2003	2 (2%)
2004	1 (1%)
2005	7 (7%)
2006	1 (1%)
2007	4 (4%)
2009	9 (9%)
2010	4 (4%)
2012	2 (2%)
2013	4 (4%)
2014	6 (6%)
2015	8 (8%)
2016	4 (4%)
2017	9 (9%)
2018	5 (5%)
2019	4 (4%)
2020	11 (11%)
2021	7 (7%)
2022	1 (1%)
2023	5 (5%)
2024	1 (1%)
ICD10 clinical area	
Certain infectious and parasitic diseases	1 (1%)
Dental services	3 (3%)
Diseases of the circulatory system	11 (11%)
Diseases of the digestive system	3 (3%)
Diseases of the ear and mastoid process	2 (2%)
Diseases of the eye and adnexa	1 (1%)
Diseases of the genitourinary system	5 (5%)
Diseases of the musculoskeletal system and connective tissue	5 (5%)
Diseases of the nervous system	8 (8%)
Diseases of the respiratory system	6 (6%)
Diseases of the skin and subcutaneous tissue	4 (4%)
Endocrine, nutritional and metabolic diseases	7 (7%)
External causes of morbidity and mortality	1 (1%)
External causes of morbidity and mortality	1 (1%)
Factors influencing health status and contact with health services	6 (6%)
Injury, poisoning and certain other consequences of external causes	2 (2%)
Mental and behavioural disorders	21 (22%)
Multiple clinical areas accessing a treatment	4 (4%)
Neoplasms	4 (4%)
Pregnancy, childbirth and the puerperium	1 (1%)
Symptoms, signs and abnormal clinical and laboratory findings	1 (1%)
Geographical region	
Multiple regions	69 (71%)
Regional	28 (29%)
Included non-UK centres	4 (4%)
Includes internal pilot trial	44 (45%)
Participant blind	
No	78 (80%)
Partially	2 (2%)
Yes	17 (18%)

**Table 8:** Trial characteristics for the 23 multi-arm trials published in NIHR journals after 2019

Characteristic, *n*(%)	Summary	
	(*N*=23)	
Multiplicity adjustment		
No adjustment, no reason provided	2 (9%)	
Not mentioned	5 (22%)	
Adjustment made	16 (70%)	
Adjustment method	Alpha of 0.01	1 (4%)
	Bonferroni	6 (26%)
	Dunnett	1 (4%)
	Hierarchical	7 (30%)
	Bonferroni (adjustment made to non-inferiority limit)	1 (4%)
Sample size multiplicity adjustment		
No adjustment	12 (52%)	
Not reported/not clear	1 (4%)	
Used alpha=0.01	1 (4%)	
Bonferroni adjustment	9 (39%)	
Confidence interval coverage		
95%	16 (70%)	
97.5%	5 (22%)	
98.3%	1 (4%)	
99%	1 (4%)	

## Abbreviations

NIHR: National Institute for Health and Care Research
RCT: Randomised controlled trial
CONSORT: Consolidated Standards of Reporting Trials
HTA: Health Technology Assessment
HSDR: Health and Social Care Delivery Research
PGfAR: Programme Grants for Applied Research
PHR: Public Health Research
EME: Efficacy and Mechanism Evaluation
GHR: Global Health Research
PRISMA: Preferred Reporting Items for Systematic Reviews and Meta-Analyses
